# Perception and Belief on Cannabinoids: A Comparative Study of Rheumatology Patients and Primary Care Physicians on the Use of Cannabinoids for Pain Management

**DOI:** 10.7759/cureus.13756

**Published:** 2021-03-07

**Authors:** Nibha Jain, Neelima Reddy Kunam, Arumugam Moorthy

**Affiliations:** 1 Rheumatology, University Hospitals of Leicester NHS Trust, Leicester, GBR; 2 Medicine, University Hospitals of Leicester NHS Trust, Leicester, GBR; 3 Medicine, College of Life Sciences, Leicester Medical School, University of Leicester, Leicester, GBR

**Keywords:** synthetic cannabinoids, complementary alternative medicine, chronic pain management, arthritis and orthopaedic rheumatology

## Abstract

Introduction

With the recent increase in popularity of cannabinoids in the management of chronic pain, the inquisitiveness among our patients and health care professionals are probably now at its peak. Many treating health care professionals in their clinical practice come across patients who either use cannabinoids or are interested in their efficacy and side effects. As there is paucity of data and research about their use in rheumatology, patient's self-reported responses and experience of primary care physicians (General Practitioners [GPs]) can guide in expanding our knowledge.

Methods

Ours was an observational, cross-sectional study among rheumatology patients and GPs in the Leicestershire area. Initial questionnaire was designed by authors addressing demographics, knowledge, experience and perception. This was piloted among patients and GPs and improvised, redesigned and used for the study. The study design consisted of two arms: first arm including GPs and second arm rheumatology patients.

Results

Arm 1 consisted of 100 GPs with median age group of 30-40 years. 34% GPs experienced their patients inquiring about cannabinoids. 78% did not believe cannabinoids benefited the patients. On a scale of 0-10, the mean benefit in managing pain 3.2 + 2.5. Arm 2 consisted of 102 patients. 16% reported using cannabinoids for managing their chronic pain. The users reported significant improvement in pain compared to non-users (p=0.002). On comparing the perception of cannabinoids between GPs and patients, there was a statistically significant difference regarding awareness and effectiveness (p<0.001).

Conclusion

With the paucity of data and research about the use of cannabinoids in rheumatology, the patient self-reported responses provided an estimate as to their efficacy. This was significantly different from the GP perception. Disease and drug-focused research is need of the hour. To our knowledge, this is the First Single Centre study in the UK evaluating GP and rheumatology patient perception on cannabinoids.

## Introduction

Recently, there has been a lot of interest among patients, general public and clinicians regarding the use of cannabinoids for the management of chronic pain. Certain forms like cannabidiol (CBD) oils are being legitimately sold in high street. Cannabinoids have gained popularity among rheumatology patients and many treating clinicians frequently come across patients who are curious about it, are using it or even asking for a prescription. They are available both legally and through alternative sources in several forms such as oil, tablets, cake and coffee among others. While research on their therapeutic indications particularly in rheumatological conditions are limited, their popularity cannot be ignored.

Cannabis plants (C. sativa) are made up of more than 100 different cannabinoids, having different effects on the body. The most common forms known are CBD and tetrahydrocannabinol (THC). THC is the component responsible for psychoactive effects while CBD is devoid of this effect [1].

The endocannabinoid signalling system encompasses cannabinoid receptors, endocannabinoids and enzymes regulating the biosynthesis and inactivation of endocannabinoids. Cannabinoids are found in three settings: endogenous ligands or endocannabinoids (arachidonic acid derivatives); exogenous plant-derived phyto-cannabinoids and synthetic tricyclic terpenes [1].

There are two primary receptors CB1 and CB2 having very different and sometimes opposite actions. CB1 is primarily expressed in frontal cortex, basal ganglia and cerebellum and is responsible for the psychotropic effects of cannabis. Overall, CB1 activation exerts an inhibitory effect on the presynaptic cell. CB2 receptors are located peripherally on immunologic and musculoskeletal cells. it’s the fine balance of effect of cannabis substance on these CB1 and CB2 receptors, that ultimately result in psychoactive or immunomodulatory effects [1]. THC is known to act more on CB1 while CBDs act on CB2 receptors.

There are several studies on the use of cannabinoids in rheumatic diseases. JWH133, a selective CB2 agonist, inhibited in-vitro production of cytokines in synoviocytes and reduced in-vivo arthritis score and bone destruction in collagen-induced arthritis (CIA) rat model [2]. Another CB2-selective agonist, HU-308, reduced synovial inflammation and joint destruction in CIA mice model [3].

Cannabis is currently a controlled drug as classified by the Misuse of Drugs Act 1971. Specific forms of cannabis are licensed for use in spasticity in adults with multiple sclerosis (MS), treatment-resistant epilepsy (Dravet and Lennox-Gastaut syndromes) and refractory vomiting secondary to chemotherapy. They require an initial prescription by a specialist medical practitioner and subsequent prescriptions may be issued by another prescriber (including General Practitioners [GPs]) as part of a shared care agreement [4]. On the other hand, use and sale of CBD products with <1 mg THC is legal in the UK [5].

Management of pain is an integral part of rheumatic diseases. Research on alternative therapies that can modulate pain and improve quality of life is essential. Paucity of data and RCT led to rejection of cannabinoids for management of chronic pain by NICE (NG144) guidelines [4].

Self-reported patient responses can assist healthcare providers and regulatory authorities in decision-making. These self-reported responses are unique indicators of impact of disease on the patient, helping in patient empowerment and necessary for determination of efficacy of the treatment. They can also be useful in the interpretation of clinical outcomes and treatment decision-making [6]. The NICE committee (2019) included the patient-reported improvement of spasticity in MS patients with the use of THC: CBD spray while licensing their use for the same indication [4].

In a self-administered questionnaire study conducted in the UK between 1998 and 2002, medicinal cannabis use was reported by patients with chronic pain (25%), multiple sclerosis and depression (22% each), arthritis (21%) and neuropathy (19%) [7]. In another self-reported prevalence of cannabinoid usage in 457 fibromyalgia patients, it was reported that 13% of fibromyalgia patients use cannabinoids, of whom 80% used herbal cannabis (marijuana), 24% used prescription cannabinoids, and 3% used both herbal cannabis and prescription cannabinoids [8].

Despite frequent discussions in the media on the use of cannabinoids as an alternative therapeutic for chronic pain, physician’s perspective and knowledge on using such therapies is still lacking in the UK. A Colorado-based survey of 114 healthcare providers concluded that though the providers frequently assess patients’ marijuana use, they are uncomfortable and inconsistent in talking to patients about specific health effects [9].

In the absence of any consistent evidence-based guidelines and recommendations for cannabinoid use in rheumatology patients, we conducted a cross-sectional study comparing self-reported responses of rheumatology patients and GPs exploring their knowledge, experience and awareness of cannabinoids use in rheumatology.

While rheumatology patients are under specialist care for their specific illness, they are ultimately under the care of their GPs who were the initial port of call by the rheumatology patients for holistic health care requirements. Therefore in our study, we chose GPs as comparative arm for more comprehensive evaluation.

## Materials and methods

This was an observational, cross-sectional study among rheumatology patients and GPs in the Leicestershire area. Initial questionnaire was designed by authors addressing demographics, knowledge, experience and perception. This was piloted among rheumatology patients and GPs which was followed by improvisation and redesigned for the use in the study.

**Figure 1:**
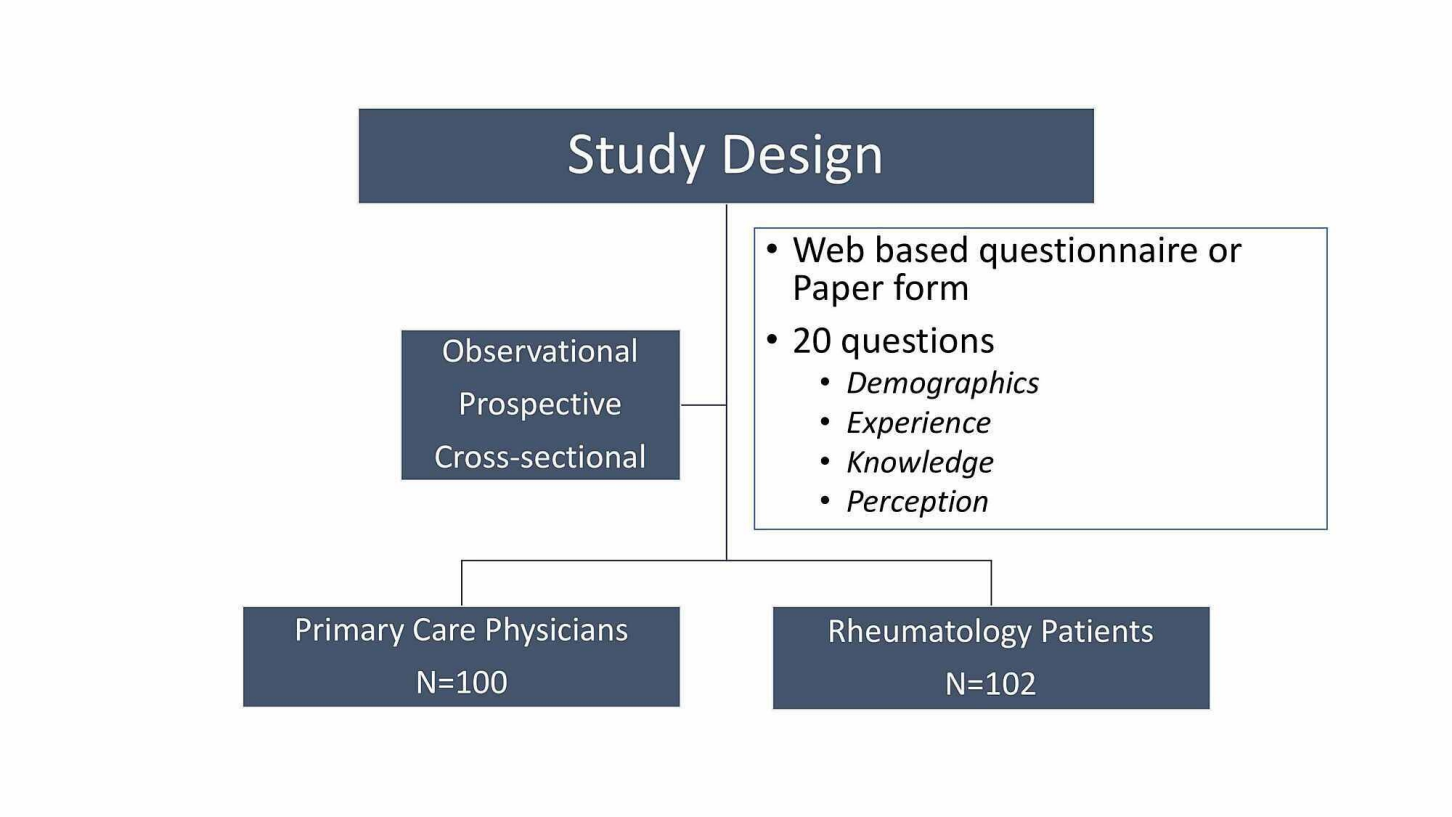
Study design.

Our study design consisted of two arms with first arm including GPs and GP trainees while second arm had rheumatology patients attending general rheumatology clinics in Leicester Royal Infirmary (Figure 1).

Questionnaires were distributed from August 2019 to February 2020. 

Arm 1: The form with 20 questions was divided into four sections covering demography, knowledge, experience and perception of cannabinoid usage for rheumatological conditions. The response was collected through web-based portal (Monkey Survey) which was sent through email. A reminder email was sent after one week. Paper-based forms were collected from GP trainees.

Arm 2: Patients were given a written participant information statement about the study and after taking informed consent and were invited to complete the anonymised questionnaire consisting of 20 questions with four sections on demography, knowledge, experience and perception of cannabinoids in managing their pain.

For grading answers, a 10-point Likert scale was used and for qualitative answers, participants were asked to rate in terms of “no help at all”, “somewhat helpful” and “extremely helpful”. 

All collected information were compiled in the Microsoft excel sheet and then analysed using SPSS Software v23.0 version (IBM Corp., Armonk, NY). Demographic data were analyzed using descriptive statistics including Chi-square test for comparing the percentage. Numerical responses were tabulated and summarized using counts and percentages. To facilitate bivariate comparisons, we collapsed qualitative and Likert scale responses to helpful and not helpful (agree or disagree) and excluded the equivocal (“somewhat”) responses.

A comparison between perception of GPs and patients regarding the efficacy of cannabinoids in managing rheumatological pain along with the opinion on availability of CBD via prescription was done. 

We also compared the experiences of cannabinoid users and non-users to shine more light in to the subject. 

## Results

Arm 1: GP response

The response rate was 70%. Total of 100 complete responses were analysed.

Section 1: Demographics

54% were male with 47% GP trainees (51% GP) and two nurses. Most of the responders (51%) had a clinical experience of up to 5 to 10 years while 21% had been practising for >15 years. Median age group was 30-40 years (Table 1).

**Table 1:** Demographic profile of participant GPs. GPs: General Practitioners.

n	100
Age group	
25-30	32
30-40	40
40-50	27
>50	1
Male:female	1.7:1 (54:46)
Current position	
GP trainees	47
GP	51
Nurses	2
Clinical experience	
0-5 years	4
5 to 10 years	51
10 to 15 years	24
>15 years	21
Number of musculoskeletal patients seen per week	
Up to 5 patients/week	25
Up to 10 patients/week	29
Up to 15 patients/week	22
Up to 20 patients/week	14
>25 patients/week	10

Section 2: Experience

While 42% of GPs were aware of complementary medicine being used by rheumatology patients, only 11% prescribed cannabinoids in their practice indicating the majority usage as off-label.

Most (58%) of the GPs replied that only 10% of their patients use any form of cannabinoids for their pain management while only 11% believed that 20% of their patients use complementary medicine for the management of chronic rheumatic pain. Only one replied 30%-50% of their patients use cannabinoids. 

Fibromyalgia (FMS) was the most common (41%) reported indication for their patients using cannabinoids, followed by rheumatoid arthritis (RA) (29%) and osteoarthritis (OA) (20%) (Table 3).

During routine clinics, 34% GPs experienced their patients inquiring about cannabinoids. When asked about the frequency of these inquiries, 4% GPs reported always, 22% reported sometimes and 8% GPs reported rarely. 15% GPs reported that patients have asked them to prescribe cannabinoids for their pain. Some of the other indications for which they have been asked to prescribe included insomnia and mood disorders. 

On asking about self-reported side effects by the patients to their GPs, 8% GPs reported adverse events which included constipation and anxiety. 

Section 3: Knowledge

Most of the GP trainees (85%) accepted that they were unaware of the indications where cannabinoids can be recommended while 57% of experienced GPs (5-10 years’ experience) were aware of the indications where cannabinoids are currently prescribed in NHS practice. 

Only 21% reported cannabinoids being used for other medical conditions which included MS, epilepsy and malignancy-related pain. Interestingly few also reported chronic pain and depression as being reasons for cannabinoid usage among their patient group.

Section 4: Perception

Most GPs (78%) did not believe that the use of cannabinoids benefited their patients much. 20% believed it did not help at all. On a scale of 0-10, the mean overall benefit they believed was 3.5 + 3.1 (median 2.5). In terms of managing rheumatological pain their response was similar with mean benefit on a scale of 0-10 being 3.2 + 2.5 (median 4.5). 

Only 14% would willingly recommend CBD usage in their practise; however, 27% agree it should be available as prescription in NHS. 

Arm 2: Rheumatology patient response

Section 1: Demographics

Total of 102 responses were analyzed where 60% were females and the majority (45%) had secondary school level education. Median age of patients using cannabinoids was 55 years. Almost half of the users were unemployed and only 33% were in full-time employment. 75% were Caucasians and 18% Asians. Very few Asian patients participated in the study and any stigma or cultural belief need to be explored in a diverse population of Leicestershire. RA (54%) was the most common diagnosis followed by OA (23%) and FMS (19%). In terms of non-rheumatological co-existent mental health diagnosis, 42% reported suffering from depression and anxiety. 

Most of the respondents (94%) complained about having ongoing pain with the majority rating pain score between 6 and 8 on a Likert scale. While 42% were satisfied with their current therapy, 18% of patients believed it was inadequate.

Of those that admitted to using cannabinoids for managing their pain (users), 88% were Caucasians and 38% reported alcohol consumption along with it. They were suffering from chronic illness with a mean duration of 6.25 years. There was no ethnicity difference noted in the use of CBD.

Section 2: Knowledge

65% of responders are aware of complementary medicine and 16% accepted using cannabinoids for their pain. 60% of the patients who did not report using CBDs were interested to know more about them. None of the responders were aware of any drug interactions or adverse effects of cannabinoids.

Section 3: Experience

Most common form of cannabinoid used was oral preparation (70%) followed by smoking or vaping (25%) with only 5% using topically. 56% reported using them for more than three months and majority used daily. RA was the most common cause of chronic pain (68%) followed by OA and FMS (25% and 24%, respectively) among users (n=16). 

All of the users had ongoing pain with a mean intensity of 7 on 0-10 scale compelling cannabinoid usage. Only one reported having experienced any side effects. 

**Figure 2:**
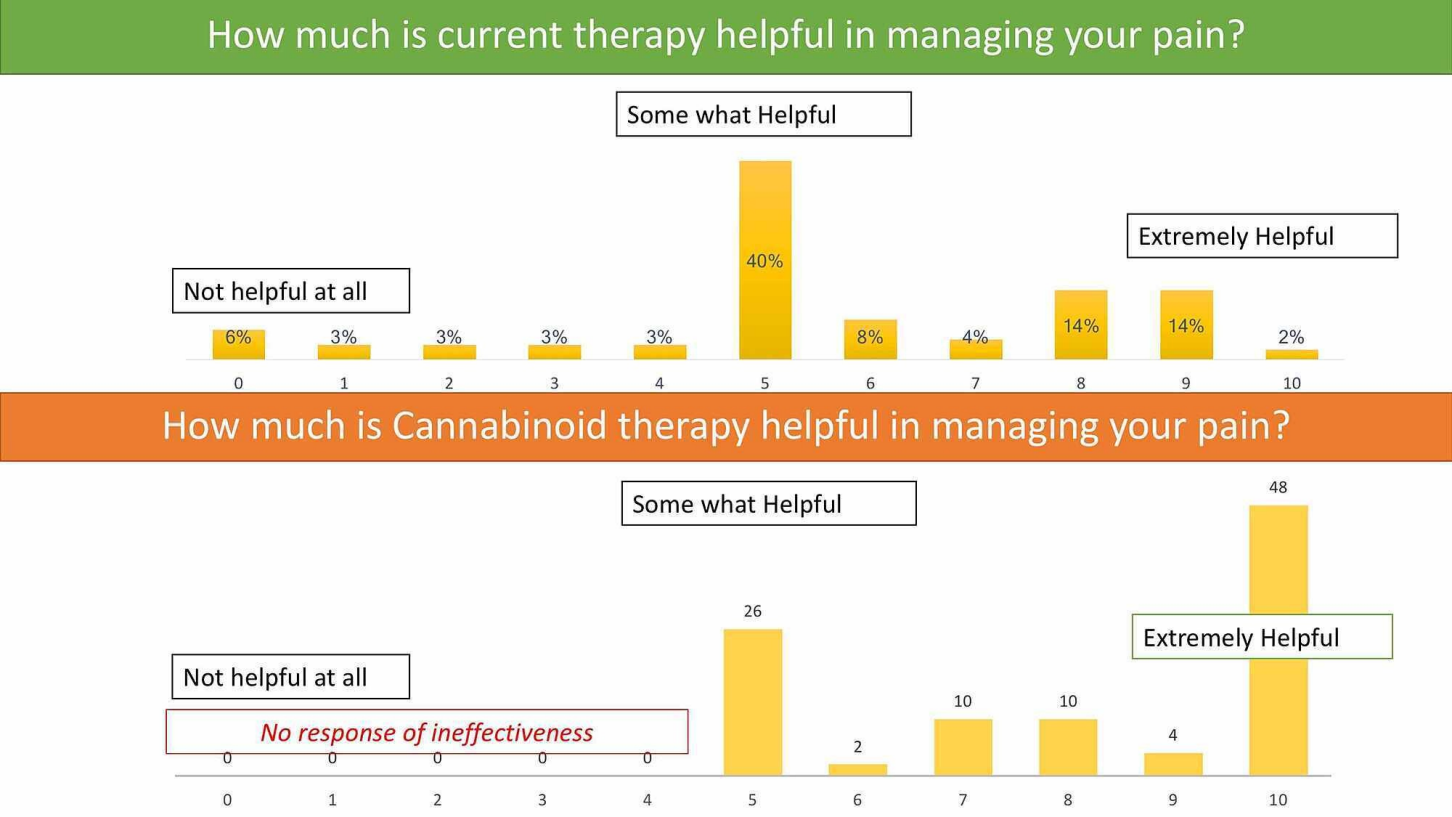
Comparison of conventional and cannabinoid use in pain management.

It was noted that while the conventional therapy was somewhat helpful in managing their pain, cannabinoids were extremely helpful with all users strongly believing that they help in managing their pain almost completely (Figure 2).

On comparing self-reported responses of cannabinoid users vs non-users there were no significant differences in terms of demography, duration of illness or nature of disease. There was a significant difference (p=0.002) in improvement in pain among those who used cannabinoids along with conventional therapy compared to those on just conventional therapy (Table 2). 

**Table 2:** Comparison of self-reported responses among cannabinoid non-users and users in a rheumatology clinic. VAS: visual analog scale.

Comparison of patient-reported outcomes (PROM) among cannabinoid non-users and users in a rheumatology clinic			
	Non-users (n=86)	Users (n=16)	p-value
Age: median (average+ SD)	55 years (54+14)	55 years (54+12)	1
Male:female ratio	2:3	1:1	0.45
Caucasians	75%	88%	0.2
Asians	19%	12%	0.5
Alcohol	39%	38%	0.94
Smoking	15%	19%	0.6
Duration of illness	7.5 years	6.25 Years	-
Diagnosis			
Rheumatoid arthritis	55%	68%	0.3
Osteoarthritis	27%	25%	0.9
Fibromyalgia	18%	24%	0.5
Depression	30%	44%	0.2
Anxiety	17%	30%	0.2
Baseline pain VAS	6	7	-
Compiled improvement from therapy (conventional vs conventional + cannabinoids)	50%	100%	0.002

Section 4: Perception

On an average, a patient spent anywhere between 2,500 and 5,000 pounds per annum on these alternative therapies but were still happy to recommend it to friends and family. Overall 49% of patients believed that cannabinoids should be available as a prescription drug in NHS while many of the non-users seemed interested to know more about it. 

Comparison of GP and patient responses

On comparing the perception of GPs and patients in pain management in rheumatology with CBDs, there was a statistically significant difference regarding the awareness and effectiveness (p<0.001). While the patients advocated the availability of cannabinoids through NHS, GPs were reluctant about it (Table 3). 

**Table 3:** Perception of cannabinoid usage in rheumatology. GPs: General Practitioners.

	Rheumatology patients, n=102	Primary care physicians, n=100	p-value
Have you heard about complementary medicine?	65%	42%	<0.001
How much does it help in pain?	8+1.5	3.2+2.5	<0.001
Would you recommend its use in?	75%	14%	<0.001
Should it be available as NHS prescription?	48%	27%	0.002
Diagnosis		*	
Fibromyalgia	19%	41%	0.001
Rheumatoid arthritis	54%	29%	<0.001
Osteoarthritis	23%	20%	0.5
Connective tissue diseases	4%	10%	0.17
*As reported by GPs			

While GPs believed that the most common indication for usage of cannabinoids was FMS (41%), patients reported RA (54%) (p=0.001).

## Discussion

To the best of our knowledge, this is one of the first studies conducted in the UK, assessing the perception of rheumatology patients and primary care physicians regarding cannabinoid usage in rheumatology. Over the last decade use of cannabinoids as complementary medicine has expanded drastically with many countries now legalizing it for various indications including pain. 

We conducted a narrative literature review and found several studies from Canada, the USA, Australia, Israel and Ireland regarding perception and experience of cannabinoids among primary care physicians and rheumatologist (Table 4).

**Table 4:** Narrative literature review of physician/rheumatologist surveys.

Survey	Survey group	Conclusion
Kondrad et al. (2011) [13]	Colorado Academy of Family Physicians	46% said that physicians should not recommend marijuana as a medical therapy at all 80% agreed that training should be incorporated into medical school curricula, 27% of those surveyed agreed that there were significant physical health benefits
Fitzcharles et al. (2013) [14]	Canadian Rheumatologists	75% lacked confidence in their knowledge of cannabinoid 45% believed there was no current role for cannabinoids in rheumatology patient care Only 25% supported any use of herbal cannabis. 70% never having previously prescribed or recommended any cannabinoid treatment
Fitzcharles et al. (2014) [15]	Rheumatology Associations of Canada and Israel	Three-quarters of responders in both countries were not confident about their knowledge of cannabinoid molecules or ability to write a prescription for herbal cannabis
Karanges et al. (2018) [16]	Australian General Practitioners	61.5% reported one or more patient enquiries about medicinal cannabis Most felt that their own knowledge was inadequate. Only 28.8% felt comfortable discussing medicinal cannabis with patients. 56.5% supported availability on prescription, with support for condition-specific use in cancer pain, palliative care and epilepsy. The majority of GPs are supportive or neutral with regards to medicinal cannabis use.
Crowley et al. (2017) [17]	Irish general practitioner	Majority did not support decriminalization but supported legalising for medical use. 60% agreed that cannabis can have a role in palliative care, pain management and treatment of multiple sclerosis (MS)
Philpot et al. (2018) [18]	Primary care providers in Minnesota-based healthcare system	58% support medical cannabis as a legitimate medical therapy 38.7% believed that providers should be offering to patients for managing medical conditions. >50%) of providers believed that medical cannabis was helpful for treating the qualifying medical conditions of cancer, terminal illness, and intractable pain
Sideris et al. (2018) [19]	New York Physicians	75% reported having patients who used cannabis for symptom control. 50% reported having patients who inquired about it. Pain was a common symptom for which cannabis was recommended by registered physicians (69%) and purportedly used by patients (83%). 84% believed opioids have greater risks than cannabinoids
Ablin et al. (2016) [20]	Israeli Society of Rheumatology	Three-quarters of responders were not confident about their knowledge of cannabinoid. 78% were not confident to write a prescription for herbal cannabis. 74% of responders held the opinion that there was some role for cannabinoids in the management of rheumatic disease

Unlike the UK, some of these states have legalised cannabinoids in pain management. The responses of these studies are in line with our findings, wherein most clinicians still do not advocate the usage of cannabinoids in chronic pain, have inadequate knowledge, less confident and have reservations regarding their legal status. On the contrary, the patient reported response from our study to support the usage of cannabinoids in rheumatic pain. 

This study raises some important concerns. There is limited confidence and lack of knowledge among GPs regarding cannabinoid usage. Furthermore, in the setting of increase demand by the patients, there is an imbalance between need, advocacy and knowledge. This serious gap covers many aspects of cannabinoids including uncertainty of content, unspecified dosing, lack of clear recommendations or guidelines and drug interactions. 

This disconnect sometimes results in difficult consultation and puts a strain on the physician-patient relationship.

Our group of patients attending regular rheumatology clinics reported using CBD more for pain in RA as compared to OA/FMS. This needs to be explored further particularly in context with possible drug interactions and failure to meet the treat to target concept [10]. 

We also noted a lack of self-reported adverse effects by the patients which could be due to appropriate safety profile or reduced concentration of THC in the preparation used. 

There was a difference in the perception of GPs regarding the use and benefits of cannabinoids in their patients compared to actual patient reported data. Possible explanation could be that GPs come across more FMS patients in their daily practice when compared to rheumatology patients. 

Strength of our study includes a good response rate and two arms comparing GPs and patient responses. The ubiquitous nature of the endocannabinoid system raises questions as to the exact function of this system in health and disease, with limited experience in the clinical arena. The cannabinoid effects are not only confined to the nervous system and pain pathways, but also have roles in inflammation and immune-modulatory effects [11]. Thus their role in the management of rheumatological issues as immune-modulators are also being explored and could be one of the reasons for their effectiveness in patients as supported by self-reported responses. 

Limitation of our study is the sample size given the popularity and abundant utilisation of cannabinoids by our patients. Although 16% of our patients reported using cannabinoids, we believe this to be under-reported and a larger study can decipher a much superior figure. In a paired survey of primary care providers(PCP) and their patients it was found that PCPs were aware of marijuana use in their patients only 53% of the time [12]. While the comparison of improvement in pain between conventional therapy and cannabinoids in the study is not ideal, it gives us an indication that those who use them are reasonably satisfied. 

 

In 2016, a Canadian study reported 3.8% of patients using cannabinoids. Most common indication (almost half) was OA. They reported users as younger, more likely unemployed or disabled, and having poorer global health [21]. Our study in contrast has a much higher prevalence of consumers (16%). Recent easy availability and access in different forms could be one of the reasons. We did not demonstrate any significant difference in terms of demography or employment status. One of the reasons for better mental health could be difference in the form of cannabinoids used among our patients. Smoking presents an uncontrolled delivery system with potential harm due to inhaled toxic substances. Sativex (CBD:THC 1:1) when administered through oro-mucosal route resulted in lower plasma levels of THC as compared with the levels achieved following inhalation at a similar dose [22]. Majority of our patients reported using oral forms which could be the reason for low psychomotor adverse events reported. Also, THC which is the component responsible for psychoactive effects is lacking in the commonly available over-the-counter preparations. 

Thus we believe the lack of knowledge among GPs stems from the paucity of clinical information regarding the use of cannabinoids in rheumatology practice. To date, there is not a single controlled clinical study that has examined the efficacy or safety of herbal cannabis in the rheumatic diseases [23]. There is a clear knowledge gap between the current science and treating clinician. This study demonstrating efficacy through patient-reported response would pave way for further studies. 

## Conclusions

Despite the lack of evidence-based medicine, rheumatology patients have access and use CBD products, sometimes without the knowledge of healthcare professionals. There is a significant difference between patient and physician perception on the use of cannabinoids as a therapeutic agent in rheumatic pain. Quality research and clinical trials are in need of the hour; however, until we have more evidence, health care professionals should currently dissuade rheumatology patients from using cannabinoids.
